# Influence of Infill Pattern on the Elastic Mechanical Properties of Fused Filament Fabrication (FFF) Parts through Experimental Tests and Numerical Analyses

**DOI:** 10.3390/ma14185459

**Published:** 2021-09-21

**Authors:** Jordi Bonada, Mª Magdalena Pastor, Irene Buj-Corral

**Affiliations:** 1 Department of Strength of Materials and Structural Engineering, Barcelona School of Engineering (ETSEIB), Universitat Politècnica de Catalunya, Avda. Diagonal 647, 08028 Barcelona, Spain; m.magdalena.pastor@upc.edu; 2 Department of Mechanical Engineering, Barcelona School of Engineering (ETSEIB), Universitat Politècnica de Catalunya, Avda. Diagonal 647, 08028 Barcelona, Spain; irene.buj@upc.edu

**Keywords:** additive manufacturing (AM), fused filament fabrication (FFF), mechanical properties, infill pattern, representative volume element (RVE)

## Abstract

Fused Filament Fabrication (FFF) is one of the most extensive additive manufacturing technologies for printing prototypes or final parts in various fields. Some printed parts need to meet structural requirements to be functional parts. Therefore, it is necessary to know the mechanical behavior of the printed samples as a function of the printing parameters in order to optimize the material used during the manufacturing process. It is known that FFF parts can present orthotropic characteristics as a consequence of the manufacturing process, in which the material is deposited layer by layer. Therefore, these characteristics must be considered for a correct evaluation of the printed parts from a structural point of view. In this paper, the influence of the type of filling pattern on the main mechanical properties of the printed parts is analyzed. For this purpose, the first parts are 3D printed using three different infill patterns, namely grid, linear with a raster orientation of 0 and 90°, and linear with a raster orientation of 45°. Then, experimental tensile tests, on the one hand, and numerical analyses using finite elements, on the other hand, are carried out. The elastic constants of the material are obtained from the experimental tests. From the finite element analysis, using a simple approach to create a Representative Volume Model (RVE), the constitutive characteristics of the material are estimated: Young’s Moduli and Poisson’s ratios of the printed FFF parts. These values are successfully compared with those of the experimental tests. The results clearly show differences in the mechanical properties of the FFF printed parts, depending on the internal arrangement of the infill pattern, even if similar 3D printing parameters are used.

## 1. Introduction

Fused filament fabrication (FFF), also known as fused deposition modelling (FDM), is one of the most popular and widely used additive manufacturing (AM) technologies in use today. This technology, like other AM technologies, was initially used for prototyping but has now evolved into manufacturing components with strength and stiffness capabilities for different end uses in various fields, especially in engineering, biomedical, automotive and aeronautics, among others [1].

In order to design any part, it is necessary to know the mechanical behavior of the material. Given their nature, i.e., layered printing, these printed materials can have anisotropic properties. On the other hand, they are non-homogeneous materials since their structure depends on the geometry and distribution of the filaments in each layer, as well as on other printing parameters. The infill pattern and raster orientation have a significant impact on the mechanical properties of the FFF samples. Several studies [2,3,4,5,6,7,8,9,10,11] investigate the influence of these parameters on the properties of FFF printed materials, mainly PLA, but also ABS. The experimental test performed in almost all cases is the tensile test.

The analysis of the mechanical properties of parts manufactured by FFF has been carried out mainly on the basis of experimental characterization [12,13,14]. The tensile test is the most important experimental test to characterize a material from a mechanical point of view. The elastic constants *E* (Young’s modulus of elasticity) and *ν* (Poisson’s coefficient) are extracted from it. From the force-elongation graph recorded during the test, the stress–strain graph is obtained, which gives the stress and strain values at the limit of proportionality, at the yield point, and at the ultimate or breaking point. There are materials that exhibit elastic asymmetry [15,16,17,18,19], in which case compression and/or bending tests will also be necessary; however, the compressive elastic modulus can be estimated from the tensile and flexural elastic modulus [19]. The following standards are generally used for plastic materials in general: ASTM D638 Standard Test Method for Tensile Properties of Plastics [20], ASTM D695 Standard Test Method for Compressive Properties of Rigid Plastics [21], and ASTM D790 Standard Test Methods for Flexural Properties of Unreinforced and Reinforced Plastics and Electrical Insulating Materials [22]; or their ISO equivalents: ISO 527, ISO 604, and ISO 178, respectively. However, due to the special properties of the 3D printed materials, it is expected that a Guide for Evaluating Mechanical Properties of Materials Made via Additive Manufacturing Processes [23] will be available soon.

Given the number of permutations that can be obtained from the possible parametric variations (type of base material, nozzle diameter, arrangement and separation of filaments in each layer, print speed, extrusion multiplier, etc.), the experimental cost of all the tests that would be necessary to be carried out is unaffordable. The material constitutive models allow predicting the mechanical properties of the printed material, as they have been validated with experimental tests of some series of specimens. Numerical analysis at the microscale allows effective prediction of the behavior of a non-homogeneous material such as FFF printed parts. A microscale representative volume element (RVE), based on cross-sectional morphology, captures the characteristics of the FDM print. Different loading states can be solved, in which the elastic constants used are those of the base material (filament) and depend on the manufacturer [24,25,26,27]. The result is the stress–strain response of the defined RVE. By homogenization, the constitutive characteristics of a heterogeneous material can be transformed into those of a homogeneous material with macroscopically equivalent “effective” mechanical properties.

Sheth et al. [28] define a representative volume cell (RVC) based on cross-sectional images. Somireddy et al. [29] calculate the elastic moduli of a layer by finite element simulation of a tensile test. Somireddy et al. [30] define two RVE models: one for the horizontal plate and one for the vertical plate. Nasirov et al. [31] use an RVE of the horizontal plate from microstructural images. Wang et al. [32] define an RVE based on an X-ray computed tomography (XCT) system to capture porosity. Anoop et al. [33] use an RVE covering one or two average voids identified from SEM. Garzon-Hernandez et al. [34] formulate a constitutive model of the continuum. They [29,31] also predict the mechanical properties using the classical laminate theory (CLT). In all cases, the experimental reference test is the tensile test, and the materials PLA and ABS.

In this paper, the influence of the infill pattern and raster angle on the tensile behavior of PLA specimens manufactured with the FFF technology is investigated. Specimens have been designed and manufactured with three different printing orientations (Flat, On-edge, and Vertical) and three patterns with different raster angles (grid, linear with a raster angle of 0° and 90°, and linear with a raster angle of 45°). The test results are analyzed, and the mechanical properties are compared as a function of infill pattern and printing orientation. From the finite element analysis, using a simple approach to create a Representative Volume Element (RVE), the constitutive characteristics of the material are estimated: Young’s Moduli and Poisson’s ratios of the printed FFF parts. These values are successfully compared with those of the experimental tests. The results clearly show differences in the mechanical properties of the FFF printed parts, depending on the internal arrangement of the infill pattern, even if the same printing parameters are used. In Section 2, the experimental tests and the computational model are reported. In Section 3, the experimental results are analyzed and compared with those estimated from the finite element model. Finally, the conclusions of the research are presented in Section 4.

## 2. Materials and Methods

### 2.1. Manufacturing of Specimens and Experimental Tests

In this work, tensile tests were performed to determine the mechanical material properties of PLA 3D printed samples according to the infill pattern and the raster angle employed in the FFF technique. All specimens were printed in white polylactic acid (PLA) filament from BCN3D Technologies. The printing equipment was a Sigma R19 from BCN3D Technologies (Gavà, Spain). The Cura software was used to obtain the G-code for the printing process. The main printing parameters are listed in Table 1.

All the parts were printed without a shell so as to obtain homogeneous structures. In order to print the specimens with a vertical orientation, printing scaffolds were required around the parts, which were removed after printing.

**Figure 1:**
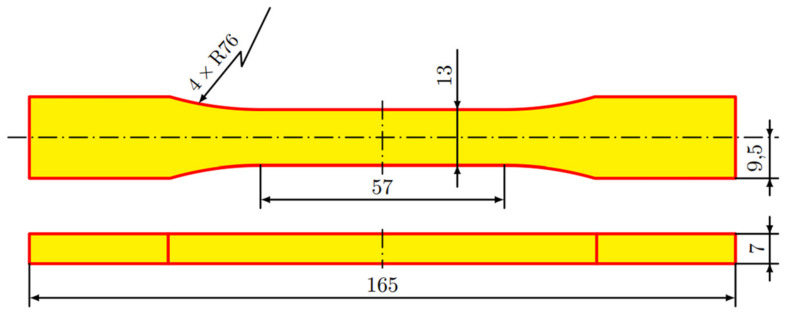
Shape and main dimensions of tensile test specimens.

**Figure 2:**
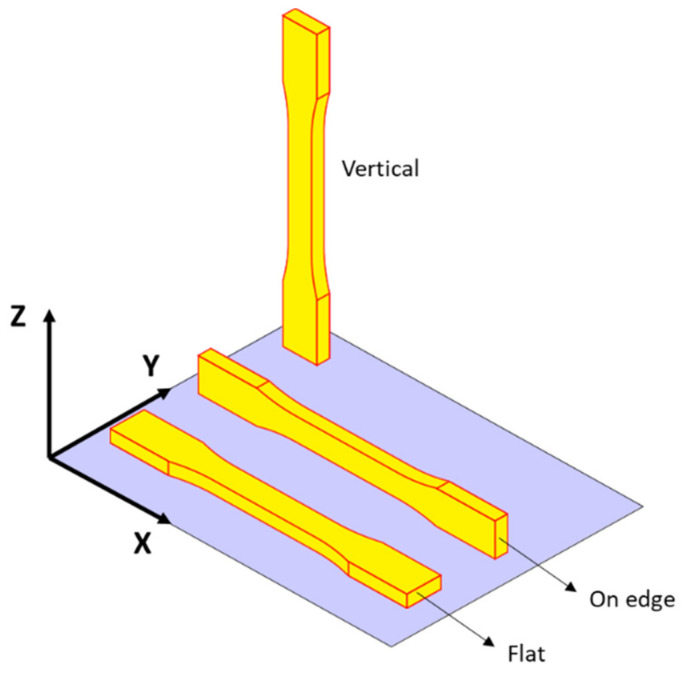
Schematics of the three different printing orientations: Flat, On-edge, and Vertical.

**Figure 3:**
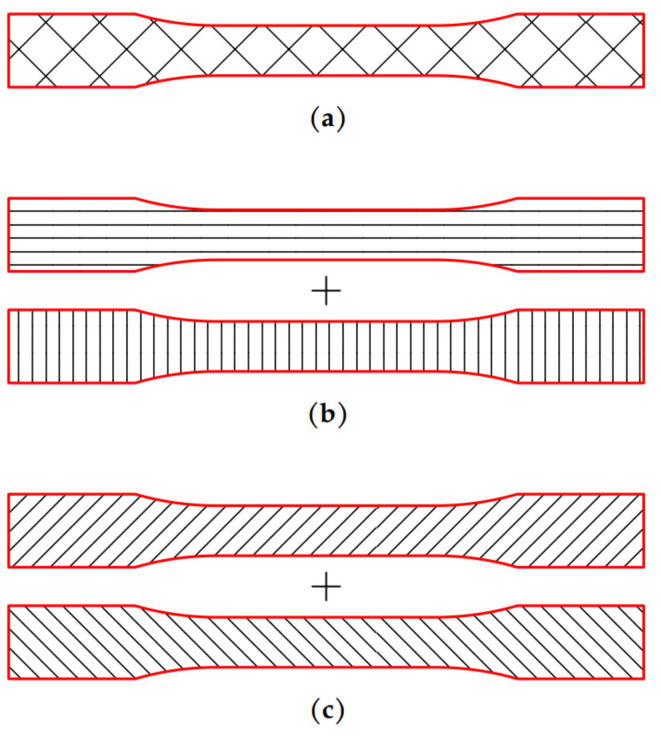
(**a**) Grid infill pattern for a single layer; (**b**) Linear090 infill pattern for two consecutive printed layers; (**c**) Linear45 infill pattern for two consecutive printed layers.

The shape and dimensions of the samples (Figure 1) were set according to the recommendations of the ASTM D 638 standard [20]. An elastic transversely isotropic constitutive material behavior was assumed as a consequence of the characteristics of the analyzed printing patterns. Therefore, 5 specimens were printed for each orientation (Flat, On-edge, and Vertical (Figure 2)) and pattern (Linear090, Linear45, and Grid (Figure 3)) to experimentally determine the main elastic parameters.

**Figure 4:**
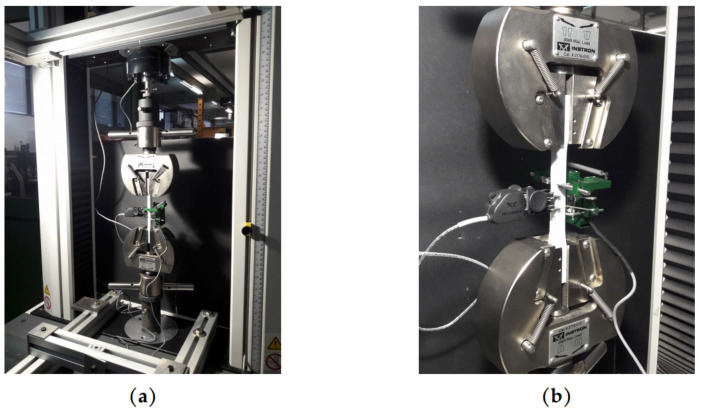
(**a**) Experimental tensile test set-up; (**b**) Details of longitudinal and transversal extensometers.

Experimental tests were conducted in an INSTRON 3366 universal testing machine (Instron, Norwood, MA, USA) with a load capacity of 10kN (Figure 4a). The longitudinal and transverse strains were measured by means of INSTRON 2630-102 and INSTRON I3574-250M-ST (Instron, Norwood, MA, USA) extensometers (Figure 4b), respectively.

### 2.2. Representative Volume Element (RVE) Model. Computational Homogenization

In this paper, PLA is used to manufacture all the samples. It is known that PLA has an isotropic behavior. Nevertheless, FFF manufactured parts commonly exhibit orthotropic characteristics as a consequence of the manufacturing process. In fact, the constitutive behavior of the printed parts depends on several printing parameters, such as infill structure or pattern, infill density, etc. When the structural behavior of an FFF printed part is analyzed through the finite element method, it is commonly considered a continuum medium in order to reduce the representation of the real geometry (internal arrangement), as well as to reduce the computational cost. Therefore, a small domain of the printed part, which represents the periodic internal arrangement of the manufactured sample, is used to create a representative volume element (RVE). The microstructure of the RVE can be analyzed using the known properties of the PLA filament to estimate the specimen macroscopic orthotropic behavior by means of a homogenization procedure.

The macroscopic constitutive relation for an orthotropic model is expressed by Equation (1):

(1) $$\left[\begin{array}{c} {\bar{\sigma }}_{11}\\ {\bar{\sigma }}_{22}\\ {\bar{\sigma }}_{33}\\ {\bar{\sigma }}_{12}\\ {\bar{\sigma }}_{13}\\ {\bar{\sigma }}_{23} \end{array}\right]=\left[\begin{array}{cccccc} C_{11} & C_{12} & C_{13} & 0 & 0 & 0\\ C_{12} & C_{22} & C_{23} & 0 & 0 & 0\\ C_{13} & C_{23} & C_{33} & 0 & 0 & 0\\ 0 & 0 & 0 & C_{44} & 0 & 0\\ 0 & 0 & 0 & 0 & C_{55} & 0\\ 0 & 0 & 0 & 0 & 0 & C_{66} \end{array}\right]\left[\begin{array}{c} {\bar{\varepsilon }}_{11}\\ {\bar{\varepsilon }}_{22}\\ {\bar{\varepsilon }}_{33}\\ {\bar{\gamma }}_{12}\\ {\bar{\gamma }}_{13}\\ {\bar{\gamma }}_{23} \end{array}\right]$$

where ${\bar{\sigma }}_{ij}$, ${\bar{\varepsilon }}_{ij}$, and ${\bar{\gamma }}_{ij}$ are the average stress and strain components calculated by averaging the local stresses and strains over the RVE volume (Equations (2) and (3)), respectively. On the other hand, *C_ij_* are the components of the constitutive matrix *C* for the macroscopic behavior of the material. The stresses $\sigma_{ij}$ and strains $\varepsilon_{ij}$ correspond to the values of the spatial position of the RVE (microscopic scale).

(2) $${\bar{\sigma }}_{ij}=\frac{1}{V_{RVE}}\int_{V}\sigma_{ij}dV$$

(3) $${\bar{\varepsilon }}_{ij}=\frac{1}{V_{RVE}}\int_{V}\epsilon_{ij}dV$$

Six independent boundary conditions can be applied to a Finite Element RVE model as a nodal displacement on two parallel RVE boundary surfaces (parallelepiped volume). Consequently, only one of the six components of the mean strain is non-null. In addition, the mean stresses can be calculated by RVE finite element analysis through Equation (2); in this way, the components of the constitutive matrix *[C]* can be obtained. More details on RVE FE models are given in Section 3.2. Finally, the compliance matrix *[S]* can be calculated by Equation (4), which allows the determination of the main elastic properties of an orthotropic model (Equation (5)).

(4) $$\left[S\right]={\left[C\right]}^{-1}$$

(5) $$\left[S\right]=\left[\begin{array}{cccccc} \frac{1}{E_{1}} & -\frac{\upsilon_{21}}{E_{2}} & -\frac{\upsilon_{31}}{E_{3}} & 0 & 0 & 0\\ -\frac{\upsilon_{12}}{E_{1}} & \frac{1}{E_{2}} & -\frac{\upsilon_{32}}{E_{3}} & 0 & 0 & 0\\ -\frac{\upsilon_{13}}{E_{1}} & -\frac{\upsilon_{23}}{E_{2}} & \frac{1}{E_{3}} & 0 & 0 & 0\\ 0 & 0 & 0 & \frac{1}{G_{12}} & 0 & 0\\ 0 & 0 & 0 & 0 & \frac{1}{G_{13}} & 0\\ 0 & 0 & 0 & 0 & 0 & \frac{1}{G_{23}} \end{array}\right]$$

In this paper, the finite element analysis of the RVE model has been performed in order to estimate Young’s moduli and Poisson’s ratios for the macroscopic behavior of the printed specimens and compare them with experimental results. Consequently, only three different finite element analyses have been performed for each RVE model (Shear moduli are not determined or compared to experimental tests). Furthermore, a transversely isotropic behavior has been assumed based on the characteristics of the infill structure (1–2 is the isotropic plane). Therefore, the compliance matrix can be simplified, as shown in Equation (6).

(6) $$\left[S\right]=\left[\begin{array}{cccccc} \frac{1}{E_{1}} & -\frac{\upsilon_{12}}{E_{1}} & -\frac{\upsilon_{31}}{E_{3}} & 0 & 0 & 0\\ -\frac{\upsilon_{12}}{E_{1}} & \frac{1}{E_{1}} & -\frac{\upsilon_{31}}{E_{3}} & 0 & 0 & 0\\ -\frac{\upsilon_{13}}{E_{1}} & -\frac{\upsilon_{13}}{E_{1}} & \frac{1}{E_{3}} & 0 & 0 & 0\\ 0 & 0 & 0 & \frac{2\left(1+\upsilon_{12}\right)}{E_{1}} & 0 & 0\\ 0 & 0 & 0 & 0 & \frac{1}{G_{13}} & 0\\ 0 & 0 & 0 & 0 & 0 & \frac{1}{G_{13}} \end{array}\right]$$

**Figure 5:**
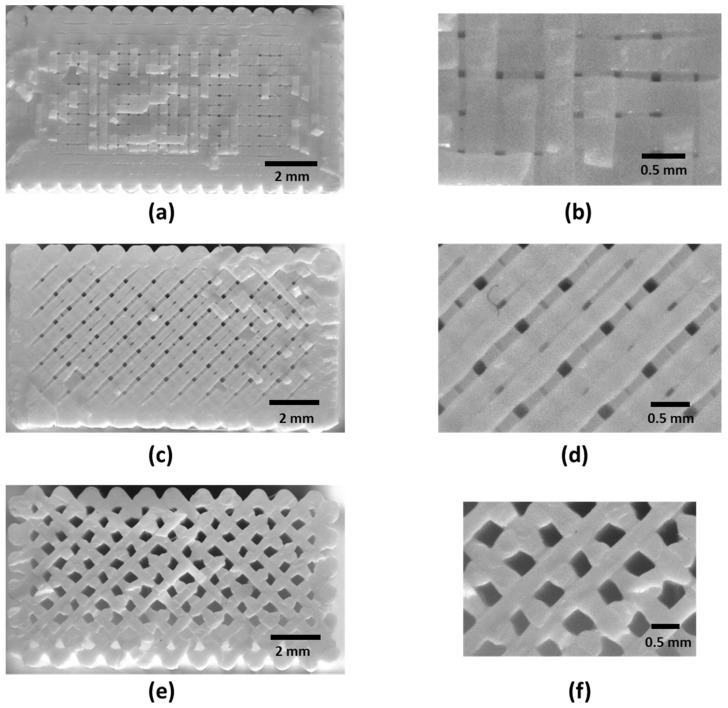
(**a**) Morphology or XY cross-section of Linear090 infill; (**b**) Morphology detail or XY cross-section of Linear090 infill; (**c**) Morphology or XY cross-section of Linear45 infill; (**d**) Morphology detail or XY cross-section of Linear45 infill; (**e**) Morphology or XY cross-section of Grid infill; (**f**) Morphology detail or XY cross-section of Grid infill.

**Figure 6:**
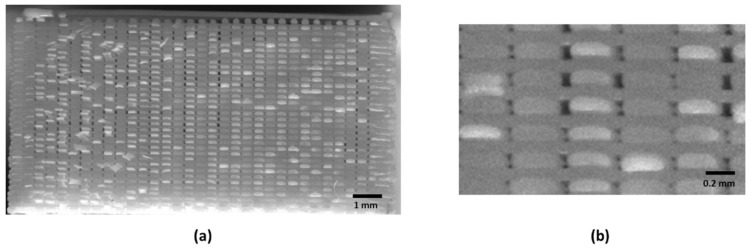
(**a**) Morphology or XZ cross-section of Linear090 infill; (**b**) Morphology detail or XZ cross-section of Linear090 infill. Air gaps between printed tracks can be observed, as well as the ratio between layer height and width of printed tracks.

A simple procedure has been established to determine the morphology (internal arrangement) of FFF printed specimens. Several images have been obtained for different samples’ cross-sections (XY and XZ). The images were taken with a 12-megapixel camera (IDS UI-2100SE-M-GL) (IDS Imaging Development Systems GmbH, Obersulm, Germany) placed close to the cross-section of the specimen (less than 150 mm). Figure 5 shows the XY morphology of Linear090, Linear45, and Grid infill patterns, respectively. The XZ morphology of the Linear090 sample can be observed in Figure 6.

## 3. Results and Discussion

### 3.1. Experimental Characterization

**Figure 7:**
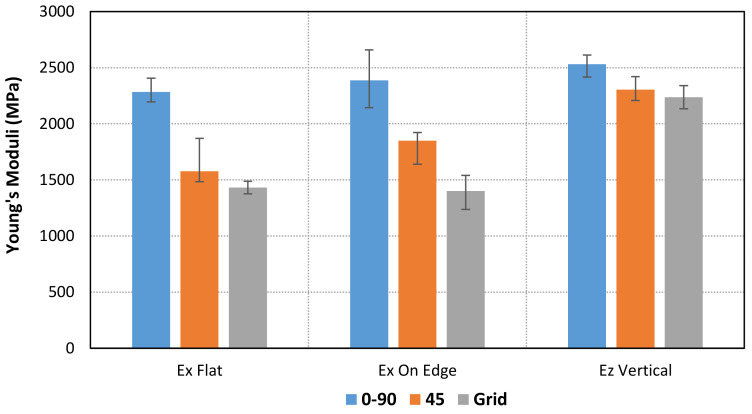
Comparison of mean values of Young’s Modulus for each orientation and pattern.

**Figure 8:**
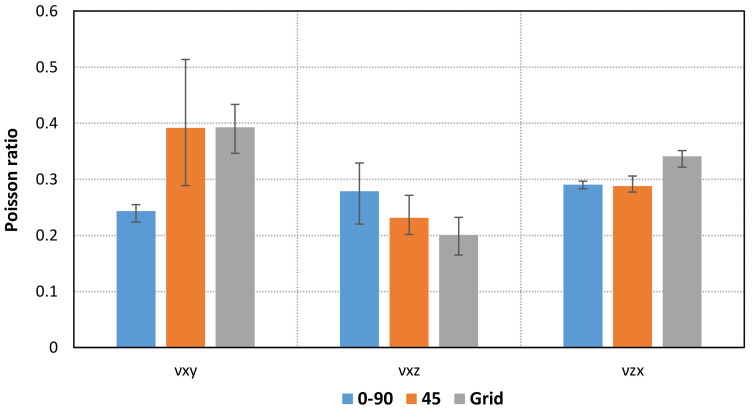
Comparison of mean values of Poisson ratio for each orientation and pattern.

Forty-five tensile tests (nine series of five units each) were performed to determine the stress–strain behavior of PLA-printed specimens based on infill pattern and orientation. Table 2 and Figure 7 and Figure 8 show the mean values of Young’s moduli and Poisson’s ratios obtained for each pattern and orientation. First of all, the results show that the maximum Young’s moduli are reached for the Linear090 infill pattern, and the minimum values correspond to the Grid pattern. In addition, similar values of Young’s modulus are obtained for Flat and On-edge orientation, as is expected, especially for Linear090 and Grid specimens. Conversely, higher differences arise for Linear45 specimens.

Initially, similar values were expected for samples with Flat and On-edge X-orientations for each infill pattern. Although the shell structure was not defined during the printing process, some differences in the specimen skin can be observed (Figure 5), which may produce differences between Flat and On-edge orientations. The results seem to indicate that the Linear45 infill pattern could be more influenced by this issue. Furthermore, it is important to point out that the internal arrangements of the Linear090 and Linear45 samples are different, as shown in Figure 5. Although the same type of infill pattern (Linear) was defined in the Cura software with a different raster angle, a variation of the distance of the printed tracks, as well as the geometry of the air voids, was obtained after the printing process.

**Figure 9:**
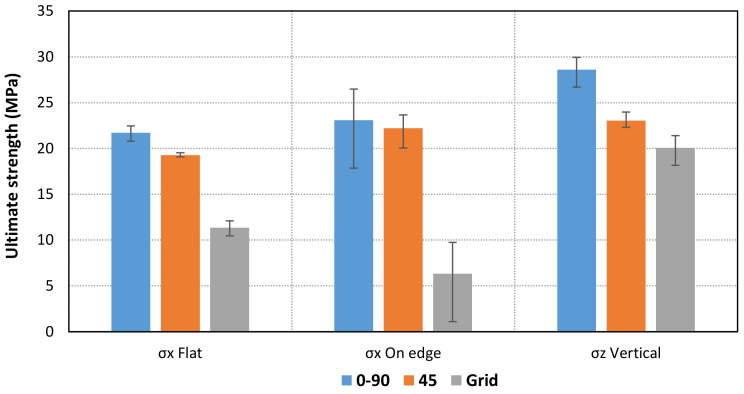
Comparison of mean values of ultimate strength for each orientation and pattern.

**Figure 10:**
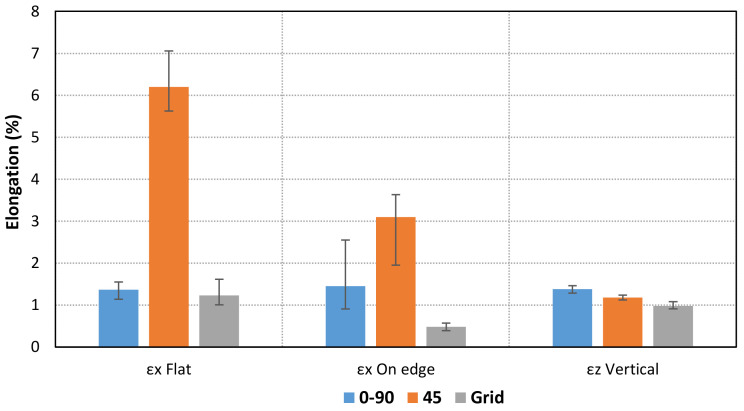
Comparison of mean values of elongation for each orientation and pattern.

**Figure 11:**
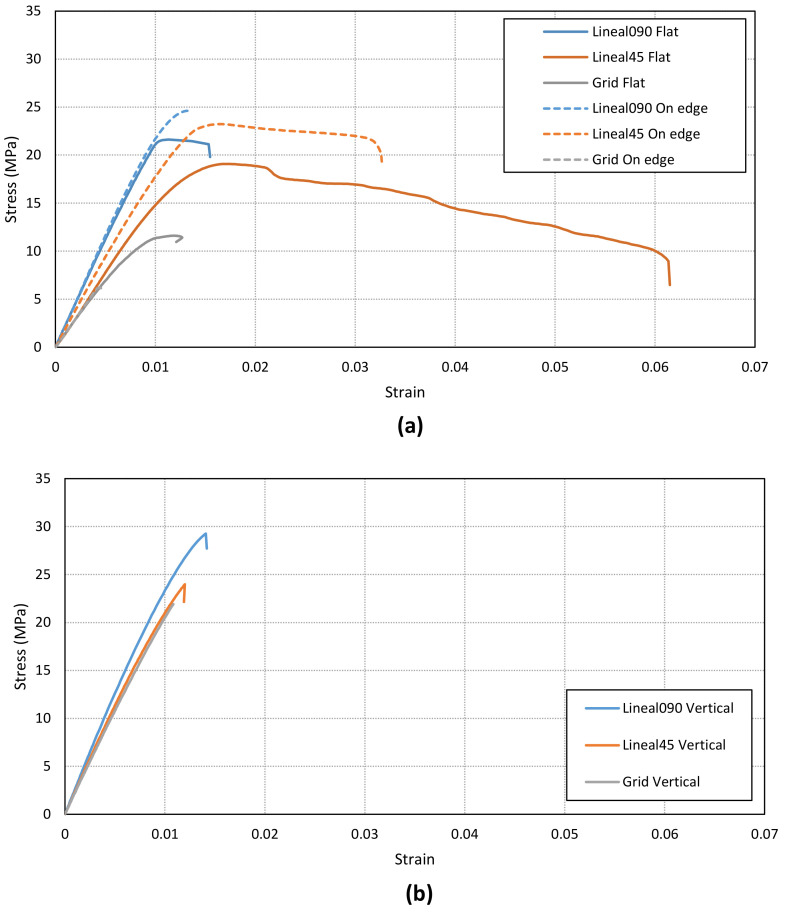
(**a**) Comparison of the stress–strain curve for Flat and On-edge orientations. The main difference is found in the Linear45 pattern; (**b**) Comparison of the stress–strain curve in the Vertical orientation.

**Figure 12:**
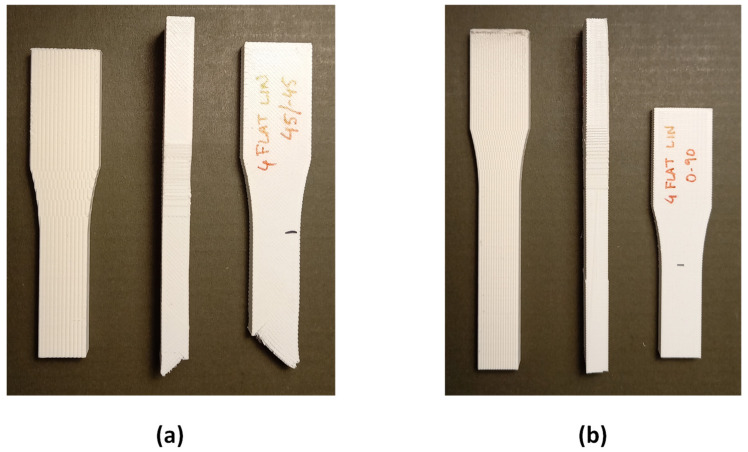
Comparison of fracture interfaces of Linear specimens: (**a**) Linear45 Vertical, On-edge, and Flat specimens (from left to right); (**b**) Linear090 Vertical, On-edge, and Flat specimens (from left to right).

In addition, the ultimate strength and maximum elongations have been calculated for each case. The specimens show different behavior depending on the infill pattern (Figure 9, Figure 10, Figure 11). The Linear45 specimens have a much more ductile behavior than the Linear090 and Grid samples. The Linear45 specimens show a different fracture interface than Linear090. In fact, the Linear45 samples show a rough fracture interface for Flat and On-edge orientations (Figure 12a), whereas for Linear090 samples, a clear plane fracture surface perpendicular to the applied axial load has been obtained (Figure 12b). This difference could produce a different ductile behavior among the Linear infill pattern specimens. Similar characteristics were found in [9]. On the other hand, all specimens (Linear090, Linear45, and Grid) with a Vertical orientation present a clear fracture surface and low elongation values.

Furthermore, Grid parts present a lower ultimate strength than the other patterns. The results also show that some differences are obtained for the Flat and On-edge-oriented specimens, especially for the Linear45 pattern.

### 3.2. Estimation of Young’s Moduli and Poisson’s Ratios by RVE Finite Element Analysis

**Figure 13:**
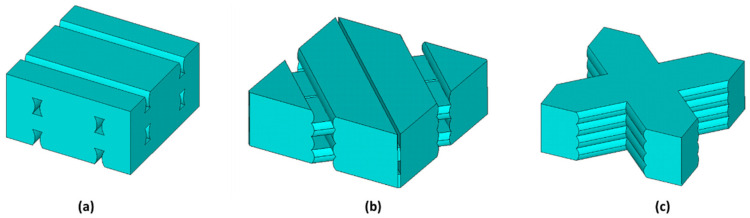
RVE geometry used for the FEA of: (**a**) Linear090, (**b**) Linear45, and (**c**) Grid patterns, respectively.

**Figure 14:**
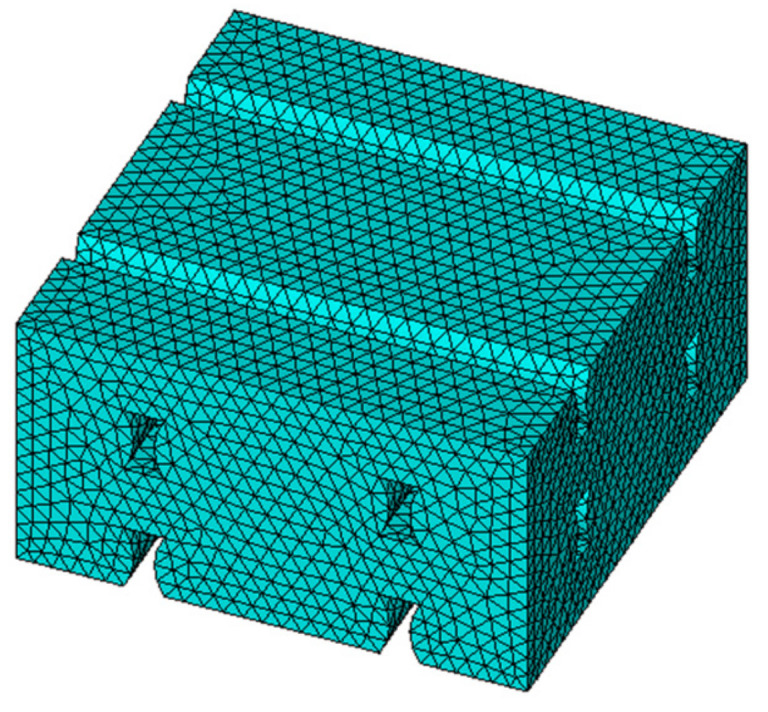
Mesh used for the Linear090 RVE model. The finite element model has 62,313 solid elements.

**Figure 15:**
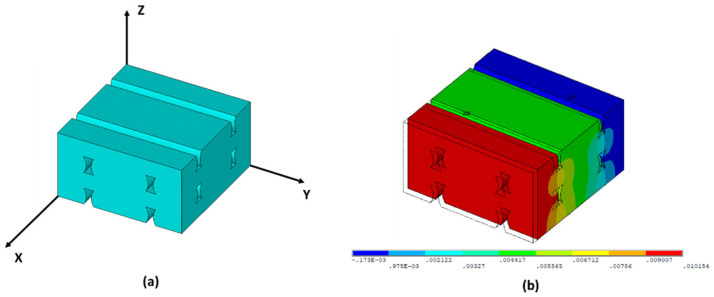
(**a**) Local coordinates used in the RVE model; (**b**) Displacement field of the Linear090 RVE model imposing a ${\bar{\varepsilon }}_{xx}\neq 0,\;{\bar{\varepsilon }}_{yy}=0,\;{\bar{\varepsilon }}_{zz}=0,\;{\bar{\gamma }}_{xy}=0,\;{\bar{\gamma }}_{xz}=0,\;{\bar{\gamma }}_{yz}=0$.

Young’s moduli and Poisson’s ratios of each infill pattern have been estimated by finite element analyses of RVE models. Figure 13 shows the RVE geometry for the Linear090, Linear45, and Grid infill internal arrangements. The relative dimensions between layer thickness, printed track width, and air voids were determined by the image analyses shown in Figure 5 and Figure 6. The FE analyses were performed in Ansys [35] 2021R1 software. Solid 185 elements were used to mesh the RVE domain. Small finite elements were used to avoid the mesh dependency in the FEA results (a minimum of 62,000 elements were used in each RVE model (Figure 14)). Three different analyses with a different set of boundary conditions were applied for each RVE model to estimate the values of Young’s moduli and Poisson’s ratios. Each set of boundary conditions applied a unique average longitudinal strain for each direction (1, 2 or 3), imposing a non-null nodal displacement of nodes located at parallel faces of the RVE domain (Figure 15).

A linear analysis was performed to obtain the stress distribution of RVE and compute the average stresses by means of Equation (2). A linear isotropic material behavior was defined for the RVE FEA with a Young’s modulus of 3120 MPa and a Poisson’s ratio of 0.36, corresponding to the values provided by the PLA filament producer. After the homogenization procedure, the compliance matrix was obtained, and the main elastic constants for the macroscopic orthotropic material were calculated. The numerical results are shown in Table 3 for Linear090, Linear45, and Grid infill patterns, respectively. In addition, the RVE density ratio (V_RVE_/V_FULL DENSE DOMAIN_) of each infill pattern was obtained and presented in Table 4.

The numerical results show that the maximum elastic moduli in the printing plane (XY) are obtained for the Linear090 infill pattern, while the minimum values are reached for the Grid pattern. On the other hand, reasonably similar values are obtained for E_Z_, although the differences between the density ratios are significant. Moreover, the main differences between Poisson’s ratios (*ν_XY_* and *ν_XZ_*) appear in the Grid infill pattern.

In addition, the numerical RVE model can also be used to estimate the value of the shear modulus, which is necessary for a complete characterization of the 3D printed material constitutive matrix.

### 3.3. Comparison of Experimental and Numerical Results

**Figure 16:**
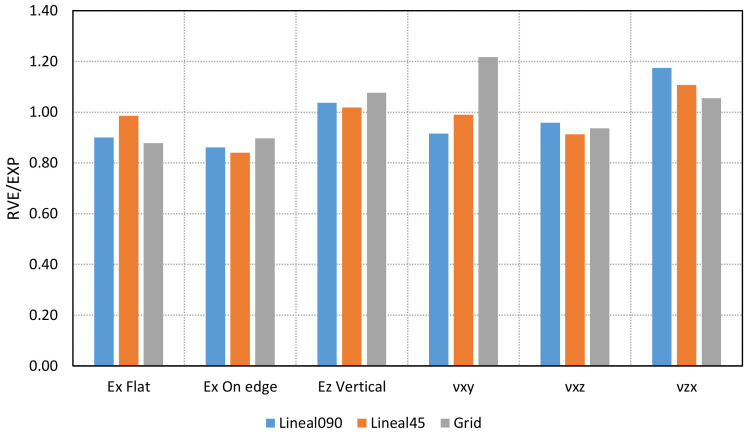
Ratios obtained between the numerical and experimental values for each of the elastic constants and each of the printing patterns.

The experimental and numerical values of Young’s moduli and Poisson’s ratios are compared in Table 5 and Figure 16.

The results show the viability to obtain a reasonable estimation of the Young’s moduli and Poisson’s ratios by means of a simply obtained numerical RVE analysis. The main differences are found in the values of Poisson’s ratios, especially for the Vertical orientation (*ν_XZ_*). Nevertheless, the numerical procedure to determine these elastic properties presents some limitations: (i) the material parameters are estimated in the linear range; and (ii) the internal pattern is the only part considered. (Some differences can be found in the skin of the specimen, although a shell structure is not defined in the printing process.)

On the other hand, the RVE approach can be perfectly used to estimate and assess the influence of the printing parameters or the infill internal arrangement. Consequently, it can be used to optimize the infill internal arrangement of a part according to its mechanical/structural requirements.

In addition, images taken with a 12-megapixel camera can be used as a simple and affordable approach to estimate the actual morphology of FFF printed parts. Table 6 shows a comparison between the theoretical density of the printed parts (% infill) and the values obtained by RVE (density obtained by image analysis) and experimental measurements on a precision scale (weight of the specimens). The results show that the actual density or infill is lower than the theoretical one defined during the printing process. Furthermore, the highest density is achieved for the Linear090 pattern, whereas the lowest is for the Grid structure. In addition, the values obtained through image analysis give values similar to those obtained with experimental weight measurements on a precision scale.

One of the main characteristics of the Grid pattern is its lower density ratio obtained after the printing process, as shown in Table 6. Consequently, some mechanical properties may be lower than those of the other infill patterns. It is important to point out that this density ratio has been obtained for the printing process defined in this paper (material, software, printing equipment, and settings) and cannot be directly extrapolated to other printing configurations.

## 4. Conclusions

This paper presents the influence of printing parameters, such as the type of infill pattern or the raster orientation, on the stress–strain behavior of the printed specimens. First, several differences have been found for Linear090 and Linear45 patterns, despite being printed with similar printing parameters, such as infill density. Moreover, higher Young’s moduli, as well as lower differences in the Poisson’s ratios, are obtained for the Linear090 infill pattern than for the Linear45 pattern. On the other hand, the Grid structure has the lowest mechanical properties (Young’s modulus, ultimate strength, and elongation). Consequently, this pattern is not recommended for printed parts with high stiffness and/or ultimate load requirements, at least for PLA material. In addition, the experimental densities are lower than the theoretical ones for all the analyzed patterns. In fact, the Grid pattern presents the lowest material density (the increase of the infill density during the printing process may result in geometrical defects in the printed samples). Finally, the Linear45 pattern presents a ductility that is clearly superior to that of the others, which can be an advantage depending on the requirements of the final printed parts.

The results also show a transversely isotropic behavior of the material for the tested samples. The highest Young’s moduli and ultimate strength are achieved for the Z specimens in all the patterns analyzed. However, these results cannot be directly extrapolated to other infill patterns or densities.

The main elastic properties of the material can also be estimated by numerical analysis of an RVE and homogenization procedure. A simple camera can be used to establish the aspect ratio between layer thickness, printed track width, and air voids to obtain an approximate morphology of the printed pattern. Consequently, the RVE model can be created without the need to use more advanced and expensive equipment measurements, such as SEM microscopy. This methodology, correctly applied, allows for a good approximation of the constitutive model of the material, thereby providing a useful tool in the design and optimization of printing patterns, with the consequent reduction of the number of experimental tests.

## Figures and Tables

**Table 1:** Printing parameters employed.

Parameter	Value
Infill (%)	99 *
Nozzle diameter (mm)	0.4
Layer height (mm)	0.1
Shell thickness (mm)	0
Printing temperature (°C)	205
Print speed (mm/s)	50
Extrusion multiplier	1
Build plate temperature (°C)	60
Type of adherence to the printing bed	Raft

* Maximum value allowed by the Cura software.

**Table 2:** Mean of main elastic constants based on infill pattern, obtained through experimental tests. The same values for E_x_ and E_y_ are assumed. (Transversely isotropic behavior is considered, as detailed in Section 2.2.)

	Orientation	Linear090	Linear45	Grid
E_X_ (MPa)	Flat	2284	1577	1431
E_X_ (MPa)	On-edge	2388	1849	1401
E_Z_ (MPa)	Vertical	2530	2304	2237
ν_XY_	Flat	0.243	0.392	0.393
ν_XZ_	On-edge	0.279	0.231	0.201
ν_ZX_	Vertical	0.291	0.288	0.341

**Table 3:** Estimation of the main elastic constants depending on the infill pattern by means of RVE finite element analysis.

	Linear090	Linear45	Grid
E_X_ (MPa)	2057	1555	1256
E_Y_ (MPa)	2057	1555	1256
E_Z_ (MPa)	2625	2346	2408
ν_XY_	0.223	0.388	0.478
ν_YX_	0.223	0.388	0.478
ν_XZ_	0.267	0.211	0.188
ν_ZX_	0.341	0.319	0.360
ν_YZ_	0.267	0.211	0.188
ν_ZY_	0.341	0.319	0.360

**Table 4:** Density ratio according to infill pattern.

	Linear090	Linear45	Grid
Density ratio	0.89	0.84	0.77

**Table 5:** Ratios obtained between the numerical and experimental values for each of the elastic constants and each of the printing patterns.

		Ratio (RVE/EXP)		
	Orientation	Linear090	Linear45	Grid
E_X_ (MPa)	Flat	0.90	0.99	0.88
E_X_ (MPa)	On-edge	0.86	0.84	0.90
E_Z_ (MPa)	Vertical	1.04	1.02	1.08
ν_XY_	Flat	0.92	0.99	1.22
ν_XZ_	On-edge	0.96	0.91	0.94
ν_ZX_	Vertical	1.17	1.11	1.06

**Table 6:** Comparison of the density ratio between theoretical values and experimental measurements according to the infill pattern.

Density Ratio	Linear090	Linear45	Grid
Theoretical	0.99	0.99	0.99
RVE (morphological measurements)	0.89	0.84	0.77
Precision scale	0.89	0.88	0.81

## Data Availability

The data are available from the author upon request.
